# Bioinformatics prediction of differential miRNAs in non-small cell lung cancer

**DOI:** 10.1371/journal.pone.0254854

**Published:** 2021-07-21

**Authors:** Kui Xiao, Shenggang Liu, Yijia Xiao, Yang Wang, Zhiruo Zhu, Yaohui Wang, De Tong, Jiehan Jiang

**Affiliations:** 1 Department of Respiratory and Critical Care Medicine, The Second Xiangya Hospital of Central South University, Changsha, Hunan, China; 2 Research Unit of Respiratory Disease, Central South University, Changsha, Hunan, China; 3 The Respiratory Disease Diagnosis and Treatment Center of Hunan Province, Changsha, Hunan, China; 4 Department of Pulmonary and Critical Care Medicine, University of South China Affiliated Changsha Central Hospital, Changsha City, Hunan Province, China; 5 Department of Pathology, The Second Xiangya Hospital of Central South University, Changsha, Hunan, China; University of Science and Technology Liaoning, CHINA

## Abstract

**Background:**

Non-small cell lung cancer (NSCLC) accounts for 85% of all lung cancers. The drug resistance of NSCLC has clinically increased. This study aimed to screen miRNAs associated with NSCLC using bioinformatics analysis. We hope that the screened miRNA can provide a research direction for the subsequent treatment of NSCLC.

**Methods:**

We screened out the common miRNAs after compared the NSCLC-related genes in the TCGA database and GEO database. Selected miRNA was performed ROC analysis, survival analysis, and enrichment analysis (GO term and KEGG pathway).

**Results:**

A total of 21 miRNAs were screened in the two databases. And they were all highly expressed in normal and low in cancerous tissues. Hsa-mir-30a was selected by ROC analysis and survival analysis. Enrichment analysis showed that the function of hsa-mir-30a is mainly related to cell cycle regulation and drug metabolism.

**Conclusion:**

Our study found that hsa-mir-30a was differentially expressed in NSCLC, and it mainly affected NSCLC by regulating the cell cycle and drug metabolism.

## 1 Introduction

Lung cancer is one of the most common cancers. Smoking and air pollution are the leading causes of lung cancer [1]. Lung cancer is also related to genetic susceptibility, and lung cancer patients are prone to familial clusters [2]. NSCLC could cause pleural effusion, chronic obstructive pulmonary disease, and pulmonary fibrosis [3]. The occurrence of NSCLC involves tyrosine kinase signaling pathway [4], mTOR signaling pathway [5], oxidative stress response [6], and cell cycle changes. The current treatments are mainly cytotoxic therapy (platinum bimodal therapy) [7]. But recently, some patients with NSCLC have developed resistance to platinum bimodal therapy.

Some studies have shown that compared with traditional treatment methods, the cure rate of lung cancer patients who have undergone gene-targeted therapy has been significantly improved [8]. In recent years, researches on molecular targeted therapy of NSCLC have made progress. These technologies have been widely used in various cancer-related research with the development of genomic sequencing and bioinformatics. MicroRNA (miRNA) is a small non-coding RNA that can regulate gene expression by interfering with the translation of the target gene [9]. MicroRNA has a unique structure and can regulate gene expression [10]. Abnormal expression of miRNA often occurs in the development of cancer. Chen et al. found out mir-148a could inhibit cancer cell migration and invasion in NSCLC (28199399). Thus, miRNA plays an important role in the treatment of NSCLC. Biometric analysis has given us a better understanding of miRNA [11]. Multiple cancer-related RNA-seq data were obtained from different databases. According to the expression of these miRNAs in cancer tissues, corresponding research strategies can be formulated. The Cancer Genome Atlas (TCGA) database has become the main database for cancer bio-information research. Hamilton et al. revealed a cancer-causing microRNA “superfamily” jointly regulated by cancer by combining the TCGA database and microRNA database [12, 13]. Li et al. also found new target genes for lung squamous cell carcinoma through gene comparison in the TCGA database and GEO database [14, 15].

In this study, we obtained all miRNAs in NSCLC tissues and paracancerous tissues from the TCGA database and GEO database. 21 meaningful differentially expressed miRNAs were identified from NSCLC tissues and adjacent tissues. The receiver operating characteristic (ROC) curve was used to analyze the sensitivity and specificity of the 4 miRNAs with the most differences, including hsa-mir-30a, hsa-mir-338, hsa-mir-451a, and hsa-mir-4732. In addition, we also performed survival analysis, gene ontology (GO) terminology, and KEGG function prediction analysis on hsa-mir-30a, which is the most differentially expressed, to determine the related expression functions and pathways of hsa-mir-30a. Differentially expressed miRNAs were identified from NSCLC tissues and paracancerous tissues. And we hope to provide a potential development direction for the treatment of NSCLC.

## 2 Methods

### 2.1. Data selection

We screened 52 NSCLC tissue specimens and 8 normal specimens from the TCGA database (https://portal.gdc.cancer.gov/). The RNA-seq data of these samples were downloaded and analyzed. Furthermore, the raw sequencing data of 5 NSCLC tissue samples and 5 normal samples were downloaded from the National Center for Biotechnology Information (NCBI) GEO database (http://www.ncbi.nlm.nih.gov/geo/) (GSE135918).

### 2.2. MiRNA screening and visualization

Bioconductor’s R language DEseq2 package was used to screen out the differentially expressed miRNAs between NSCLC tissues and normal tissues. All differentially expressed miRNAs were shown in volcano maps. And |logFC| >2 and P<0.05 were used as standards to identify differentially expressed miRNAs through the “limma” R software package. Venn diagram software was used to identify co-expressed differentially expressed miRNAs in all databases. The differential miRNAs shared in the TCGA database and GEO database were screened out. A heatmap displayed differentially expressed miRNAs between NSCLC tissues and normal tissues. Red represents highly expressed miRNA, and blue represents low expressed miRNA. 4 miRNAs with different expression between NSCLC tissues and normal tissues were selected to make boxplots that could display their expression levels.

### 2.3. ROC diagnosis and survival curve

The data were summarized into Graph Pad Prism 8.0 (GraphPad Software, Inc., San Diego, CA, USA) to make ROC curves and calculate the AUC (Area Under the ROC Curve). MiRNAs with AUC>0.8 can be used as diagnostic indicators. The most differentially expressed miRNAs in NSCLC and paracancerous tissues were selected for survival analysis. Kaplan Meier plotter (http://www.kmplot.com) was explored to analyze miRNA survival curves. According to the median value, gene expression was divided into high expression and low expression. Then, We utilized the Kaplan-Meier method and Log-rank test to evaluate the differences in patients’ survival between the high-risk and low-risk groups. P-value <0.05 representing significance.

### 2.4. GO and KEGG pathway enrichment analysis

The R language package (clusterProfiler 3.14.0) (http://bioconductor.org/packages/release/bioc/html/clusterProfiler.html) was performed to reveal the GO term and KEGG pathway analysis on miRNAs with significant survival differences. Go term is a database that can be used to define and describe the functions of genes and proteins in various species. It can be used to describe the role of genes and proteins in cells, so as to comprehensively describe the properties of genes and gene products in organisms. We used the Go term to find our target miRNA mainly related to the cellular component (CC) and molecular function (MF) levels. In addition to the annotation of the gene’s function, the gene also participates in various pathways of the human body. The database formed based on the pathway of the human body is the database related to the pathway. The top 15 pathways were selected with high gene enrichment in the KEGG pathway for analysis. P <0.05 was considered significant.

## 3 Results

### 3.1. Screening of miRNAs differentially expressed in NSCLC

**Fig 1:**
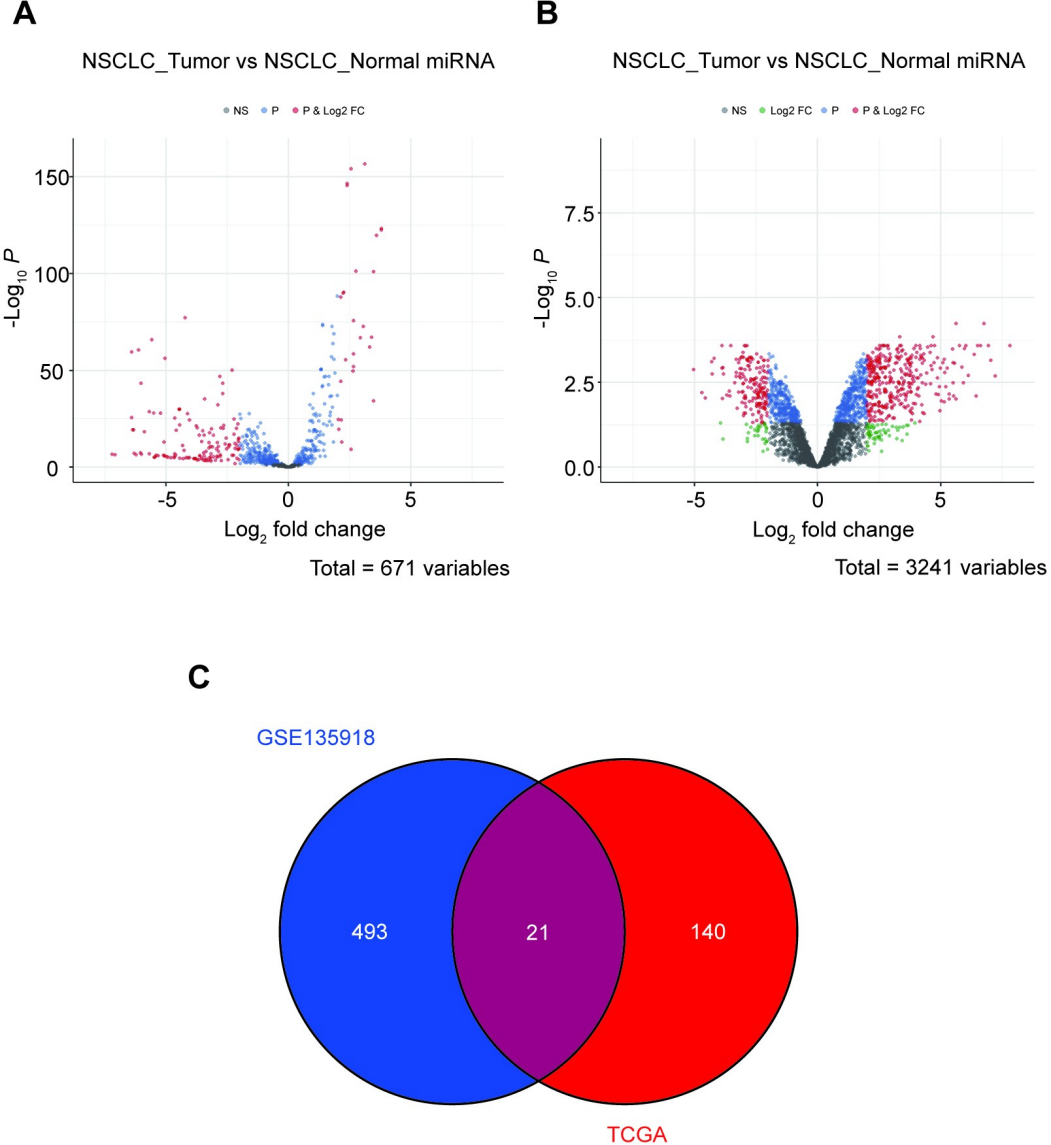
Screening of miRNAs shared by TCGA database and GEO database. A: The volcano map of miRNAs differentially expressed in NSCLC screened under the TCGA database. (P>0.05). B: Screening differentially expressed miRNA volcano maps in NSCLC under the GSE135918 database (p>0.05). The red dots on the volcano map represent differential miRNAs. C: Venn diagram of intersection of TCGA and GSE135918, the numbers represent the number of miRNAs.

We screened differentially expressed miRNAs in the TCGA database and GEO database (GSE135918) and produced volcano maps (Fig 1A and 1B). We screened a total of 671 miRNAs from the TCGA data set (Fig 1A). A total of 3241 miRNAs were screened from the TCGA database (Fig 1B). |logFC| >2 and P<0.05 were the criteria, we screened out miRNAs with statistical significance. Venn diagram showed that there were 21 common differentially expressed miRNAs in the two databases (Fig 1C). And we selected these 21 miRNAs for follow-up research.

### 3.2. The expression of miRNA in NSCLC

**Fig 2:**
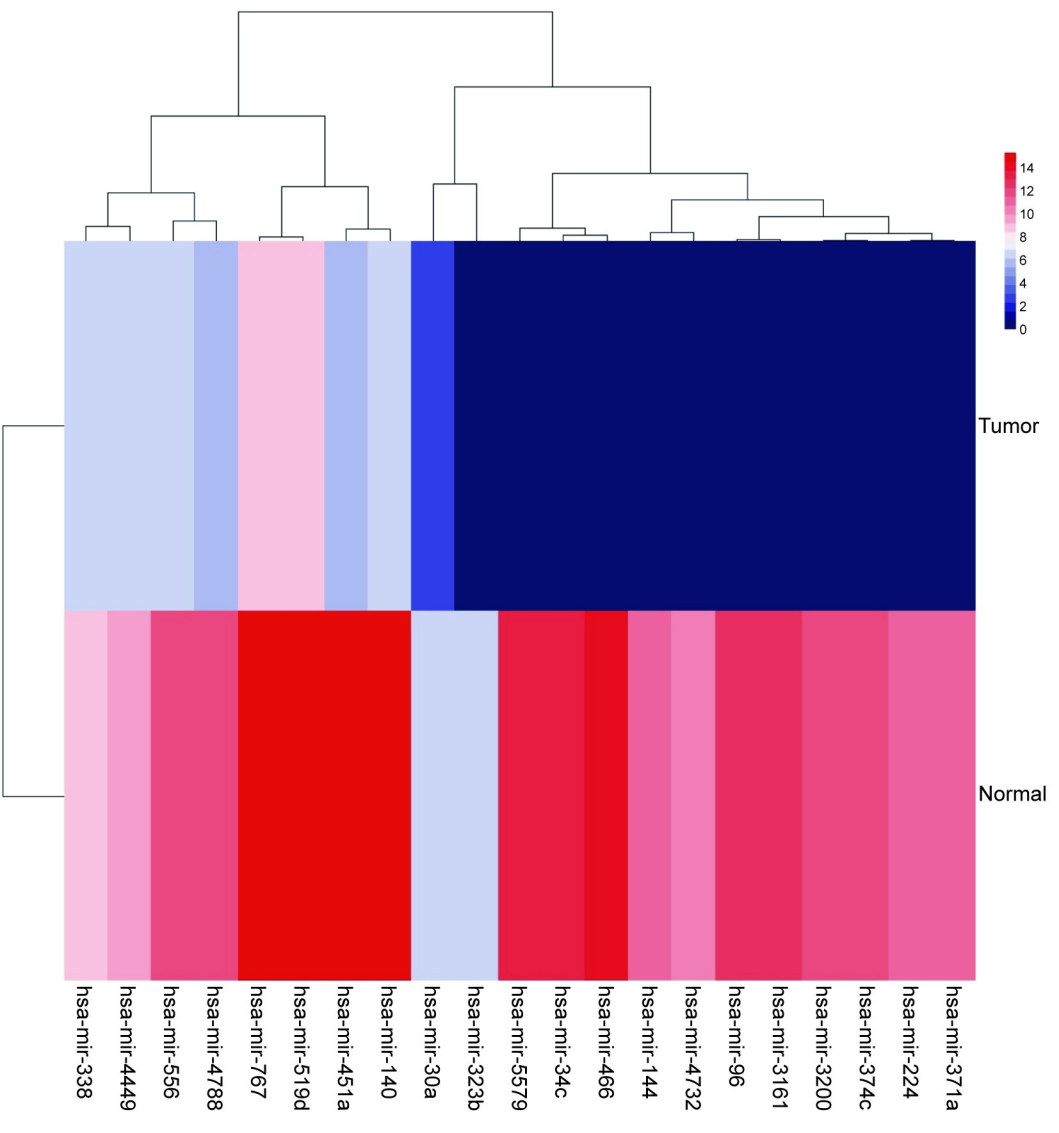
MiRNA clustering heat map showed the expression of 21 miRNAs. The abscissa represents the differential miRNA. The ordinate represents the sample, Normal, Tumor: NSCLC tissue. The color scale represents the abundance of gene expression, p<0.05.

The 21 common miRNAs screened out in the two databases (Fig 1C) were made into cluster heat maps. In the heat map, the redder indicated, the higher the miRNA expression. The expression of these miRNAs in normal tissues is higher than that in cancer tissues. Therefore, we could reasonably speculate that NSCLC development may be affected by the low expression of these 21 genes (Fig 2).

**Fig 3:**
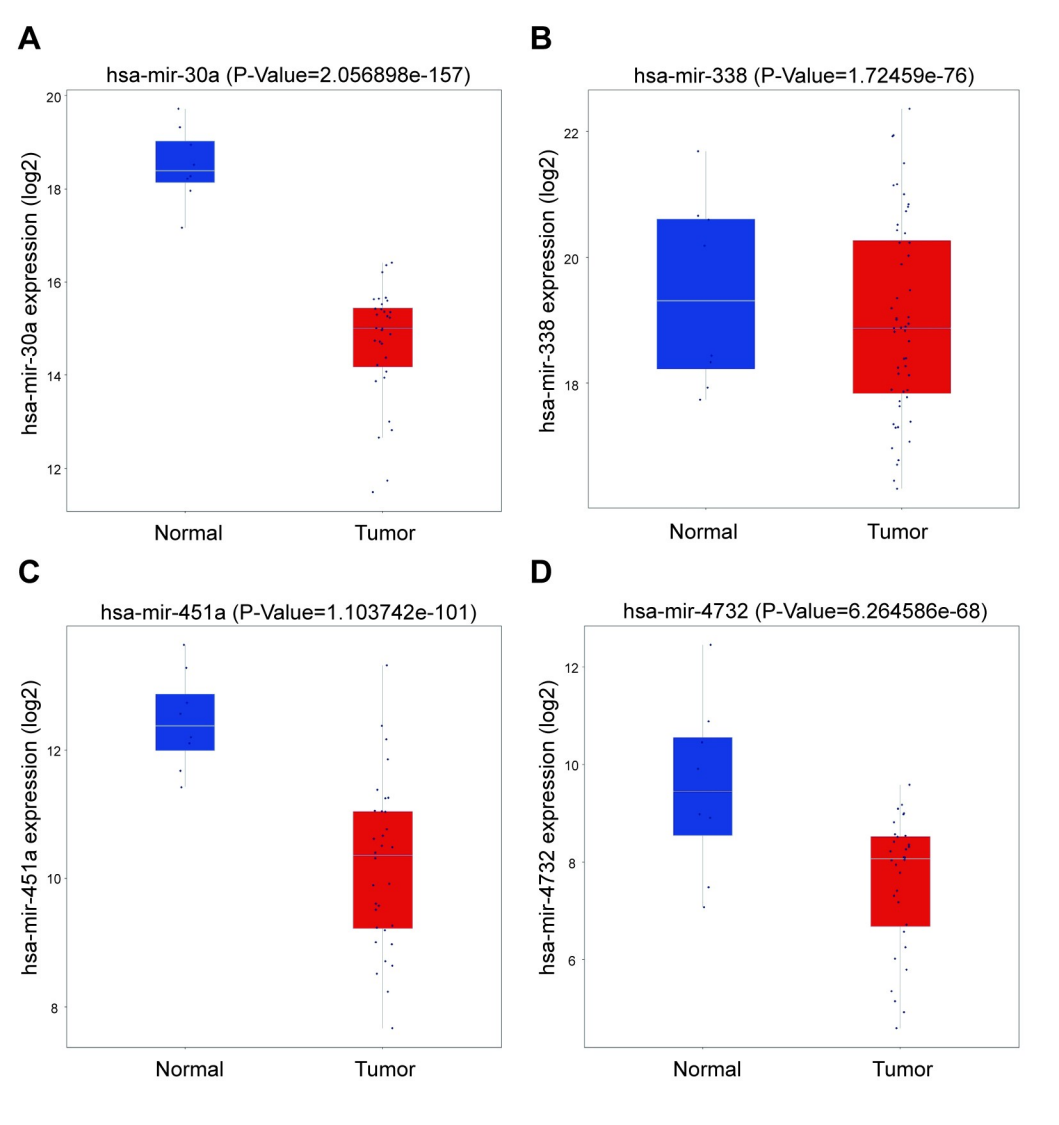
MiRNA expression in boxplots. A: Boxplot of hsa-mir-30a. B: Boxplot of hsa-mir-338. C: Boxplot of hsa-mir-451a. D: Boxplot of hsa-mir-4732. The abscissa represents the sample, Normal: Paracancerous tissue, Tumor: NSCLC tissue. The ordinate represents the expression level of miRNA, p<0.05.

From these 21 miRNAs, we screened out 4 miRNAs that have not been studied by previous people and differed significantly in NSCLC tissues and paracancer tissues. We made their expression in NSCLC tissues and para-cancerous tissues. The box diagram (Fig 3).

**Fig 4:**
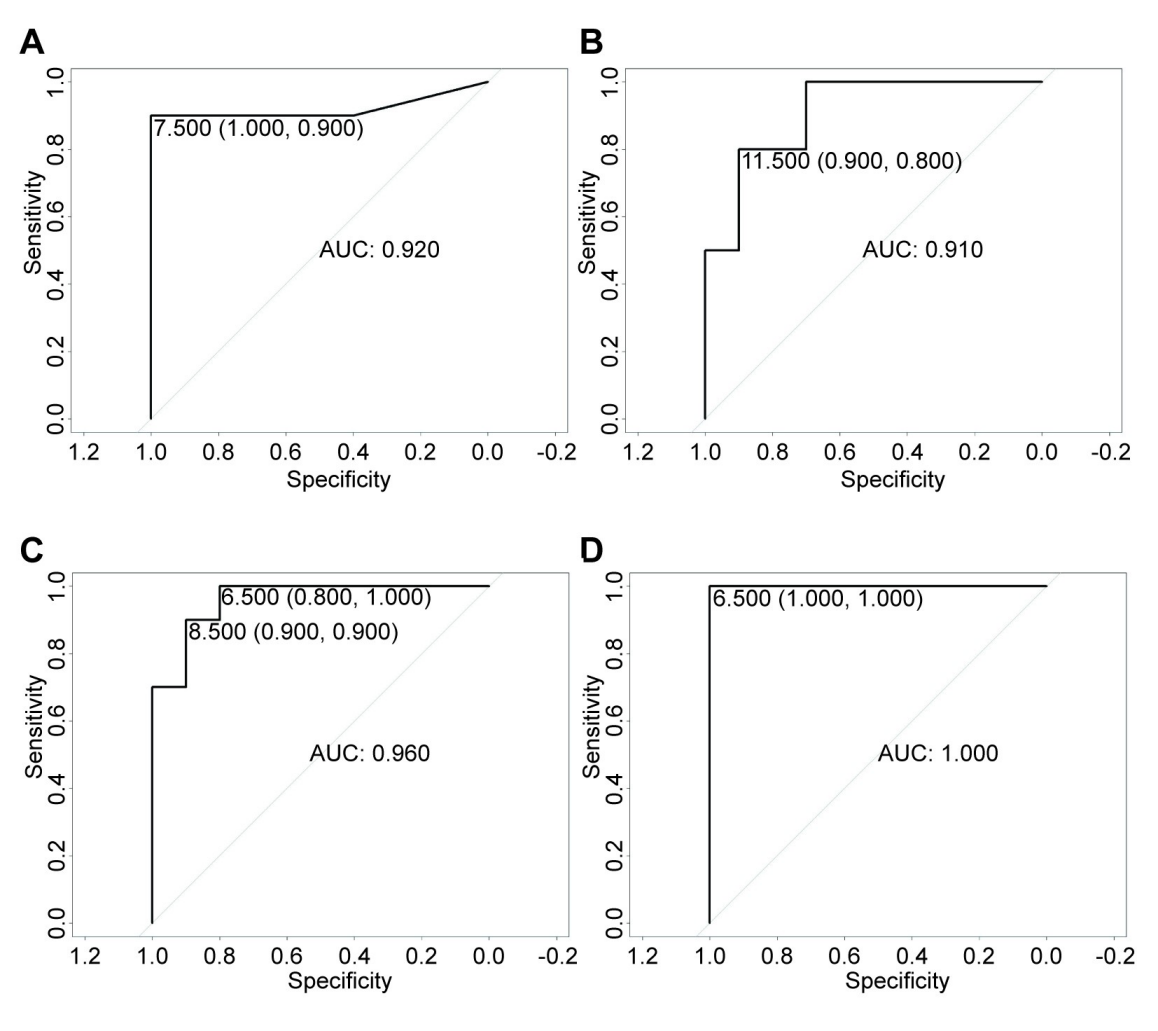
ROC analysis of miRNA. A: ROC analysis hsa-mir-338. B: ROC analysis hsa-mir-30a. C: ROC analysis hsa-mir-451a. D: ROC analysis hsa-mir-4732. The AUC>0.8 of the selected miRNA indicates that the result is meaningful.

Next, we performed ROC analysis on the selected 4 miRNAs. The results showed that hsa-mir-30a (AUC = 0.91), hsa-mir-338 (AUC = 0.92), hsa-mir-451a (AUC = 0.96) and hsa-mir-4732 (AUC = 1) were related to NSCLC (Fig 4).

### 3.3. Survival prediction of has-mir-30a

**Fig 5:**
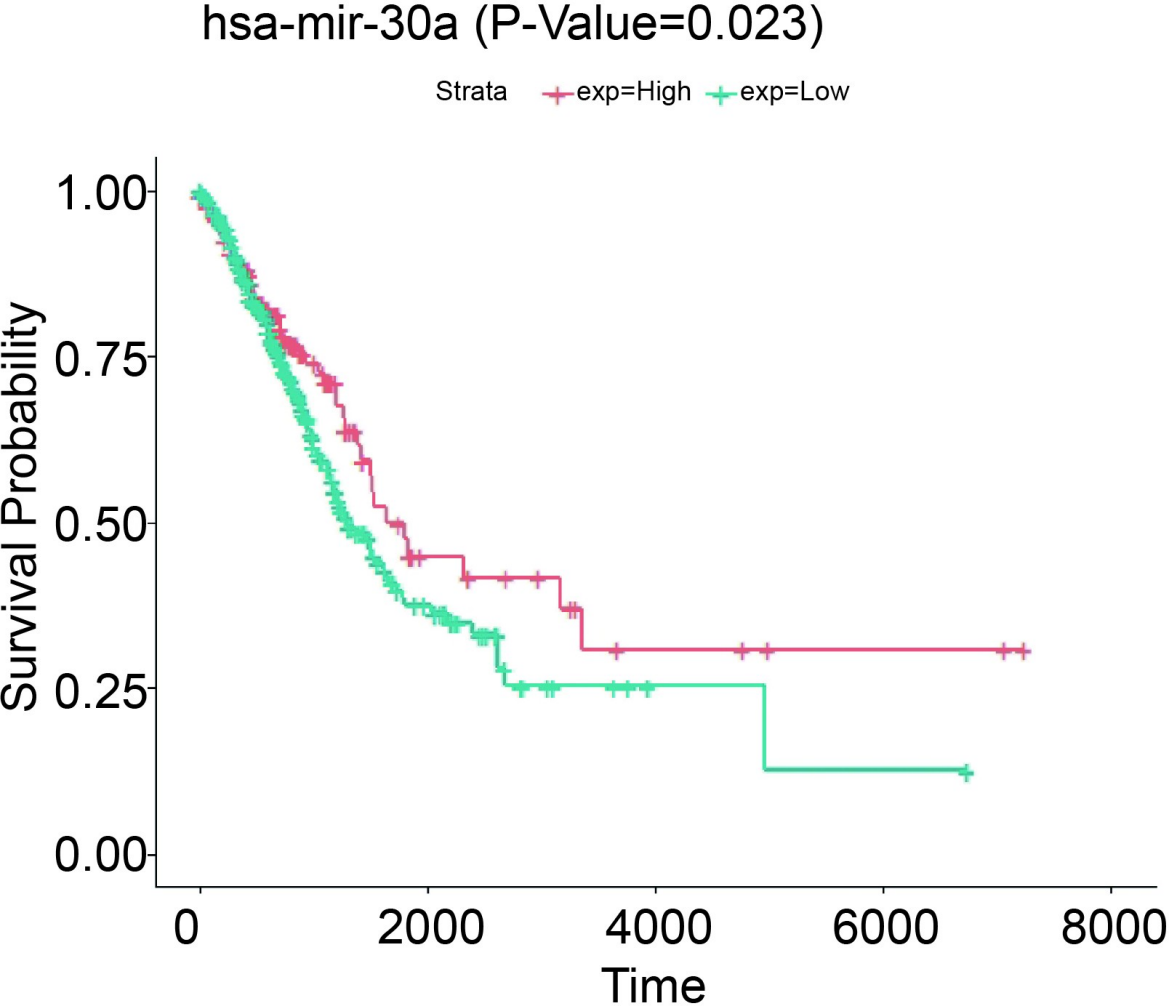
Survival analysis of differentially expressed hsa-mir-30a. The abscissa and ordinate represent respectively survival time (days) and survival rate. The blue and red lines represent respectively the low-risk and high-risk curves, p<0.05.

To explore the effect of the differential expression of miRNA on the survival rate of patients, hsa-mir-30a was selected to make survival analysis predictions. The analysis results (Fig 5) showed that compared with the high expression of hsa-mir-30a, the patients’ survival rate with low expression was significantly lower. The prediction results showed that the survival time of patients with low has-miR-30a expression was significantly lower than that of patients with high expression in NSCLC. Therefore, it was speculated that the expression of hsa-mir-30a could impact the survival rate of NSCLC patients.

### 3.4. The function prediction analysis of has-mir-30a

**Fig 6:**
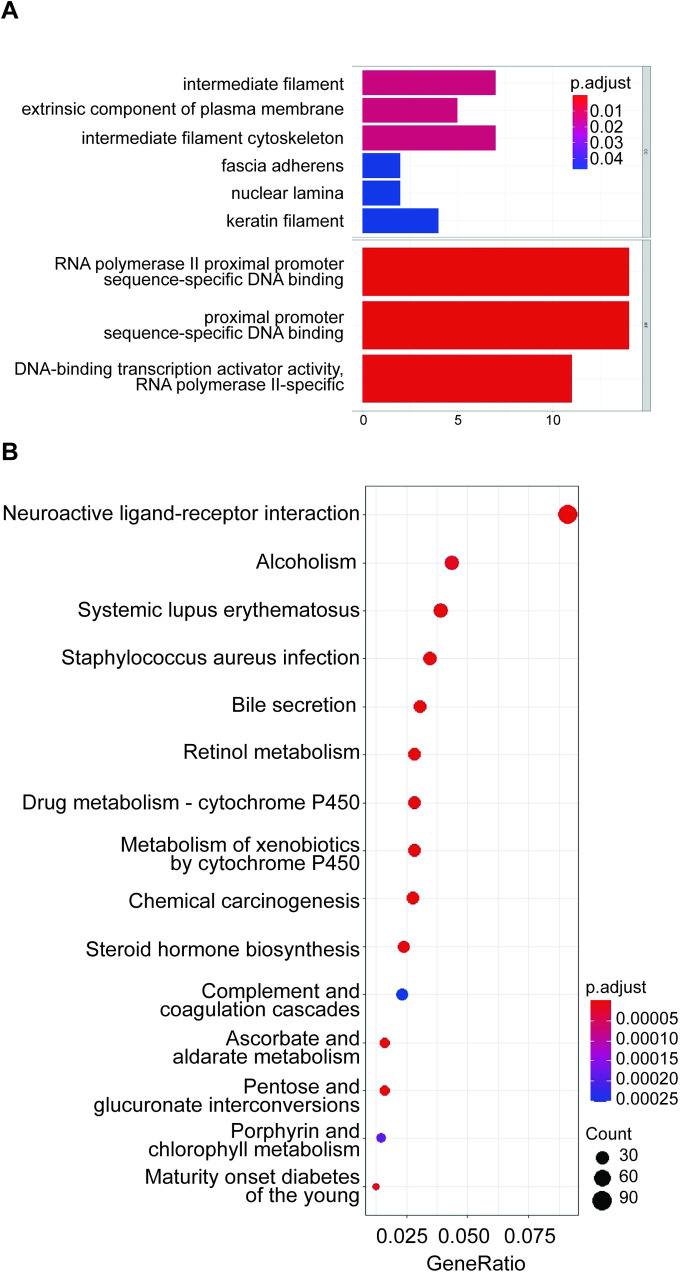
GO term and KEGG pathway regulated by hsa-mir-30a related mRNAs. A: GO term function prediction. The ordinate represents GO term description information, CC: cell composition, MF: molecular function, the abscissa is the number of differential genes enriched to the term, p<0.05. The color of the P-value ranges from blue (0.04) to red (0.01). B: KEGG function prediction. The ordinate represents gene-related pathways, the abscissa represents Gene Ratio, and the size of dots represents the number of genes. The color of the P-value ranges from blue (0.00025) to red (0.00005). The smaller the P-value, the higher the enrichment of the KEGG function.

GO term and KEGG pathway enrichment analysis were used to predict the related mRNA function of hsa-mir-30a (Fig 6). In cell components (CC), hsa-mir-30a’s operations were mainly concentrated on intermediate filaments, the extrinsic component of the plasma membrane, and the intermediate filament cytoskeleton (Fig 6A). In molecular function (MF), the genes were primarily related to RNA polymerase II proximal promoter sequence-specific DNA binding, DNA-binding transcription activator activity, and RNA polymerase II-specific (Fig 6A). The KEGG pathway shown that the genes were related to cytochrome P450 drug metabolism and heterogeneous metabolism (Fig 6B). On the one hand, The GO term of hsa-mir-30a was mainly concentrated in the nuclear layer, intermediate filaments, and RNA polymerase II. We speculated that hsa-mir-30a-related mRNAs could regulate the cell cycle process of NSCLC cells. On the other hand, the KEGG pathway of hsa-mir-30a mostly had involvement with cytochrome P450. Therefore, we conjectured that the function of hsa-mir-30a might be relevant to drug metabolism.

## 4 Discussion

NSCLC has a high incidence, and traditional treatment methods have caused drug resistance. Thus, there is an urgent need to find effective treatments for NSCLC. We used bioinformatics analysis, such as ROC analysis, survival curve analysis, and GO term and KEGG pathway functions, to select NSCLC related miRNAs. We hope they could provide a scientific basis for the treatment of the disease. We used the bioinformatics analyses to show the correlation between hsa-mir-30a and NSCLC for the first time. The low expression of hsa-mir-30a reduces the survival rate of patients. At the same time, we also predicted the pathways related to hsa-mir-30a, that is, the regulation of cell cycle and tumor resistance. These results suggest that HSA miR-30a may be an effective target for NSCLC treatment.

In recent years, some calculation methods for predicting the potential association between miRNA and disease have received widespread attention. Chen et al. used a new matrix model-Inductive Matrix Completion for MiRNA-Disease Association prediction (IMCMDA), to predict the association between various tumors and the first 50 DEGs [16]. Furthermore, Chen et al. proposed a new model, Ensemble of Decision Tree based MiRNA-Disease Association prediction (EDTMDA). This model can analyze miRNA and disease pairs by calculating statistical measures, graph theory measures, and matrix decomposition results and comprehensively consider the correlation between miRNAs and various diseases [17]. Chen et al. also proposed another model, Neighborhood Constraint Matrix Completion for MiRNA-Disease Association prediction (NCMCMDA), which combines neighborhood constraints with matrix completion and uses similar information to predict the association between miRNA and disease [18]. By comparing 20 different calculation models, Chen et al. found that each model has its advantages and disadvantages [19]. In short, the main advantage of bioinformatics-based models is that they can predict potential miRNAs for new diseases and significantly save experimental costs [19]. However, the conclusions drawn through bioinformatics analysis may not necessarily apply to the clinic. We still need a large number of experiments to verify our findings. Our work analyzes and compares the most differentially expressed miRNAs in non-small cell lung cancer and paracancerous tissue samples in the two databases and analyzes its two-year survival rate and related pathways. At the same time, according to the specificity and sensitivity of the differentially expressed miRNAs, the AUC value was calculated. The statistical method we use is linear regression. We calculated that the AUC value of the has-mir-30a-NSCLC pair is 0.92. It can be explained that there is a correlation between has-mir-30a and NSCLC.

Furthermore, the mutual regulation between lncRNAs and miRNAs is related to the occurrence of various diseases. Zhang et al. constructed the LMI-INGI model and calculated the scores of lncRNA-miRNA pairs based on the similarity construction map of lncRNA and miRNA to judge the correlation between the two [20]. Liu et al. used the LMFNRLMI model to calculate the association of lncRNA-miRNA pairs [21]. miRNA-lncRNA interaction prediction is very valuable for NSCLC. We are also interested in miRNA-lncRNA interaction prediction. In the future, we will study the interaction prediction of miRNA-lncRNA, the mechanism of action, the signal pathways involved, and the relationship between miRNA-lncRNA and apoptosis.

We selected differentially expressed RNA-seq data in the TCGA database and GEO database. As a result, 21 differentially expressed miRNAs shared in the two databases of samples were obtained. After a literature search, some of these 21 miRNAs have been involved in cancer-related gene-targeted therapy research. Among them, hsa-mir-4732 (AUC = 1) has been used in the lifespan research of lung adenocarcinoma, and it has been found to have significant transcription disorders [22]. Analysis of differentially expressed miRNAs in peripheral blood of patients with NSCLC found that the expression of hsa-mir-4449 (AUC = 1) was significantly down-regulated [23]. But in NSCLC, the specific role of these miRNAs needs further verification.

Our results showed that the expression of hsa-mir-30a in NSCLC tissues was significantly lower than in the normal. Compared with other miRNAs, the difference of hsa-mir-30a was the most significantly. Li et al. analyzed the miRNA genes in SCLC by using bio-information technology in the GEO database and found that hsa-mir-30a was down-regulated in SCLC [24]. Wang et al. found that miR-30a-3p was significantly reduced in gastric cancer tissues than normal tissues through bioinformatics analysis [25]. However, the expression of has-mir-30a has not been detailed reported in NSCLC. Our results show that there is a strong association between has-mir-30a and NSCLC. Next, we performed the survival analysis of hsa-mir-30a and the function prediction analysis of related mRNA of hsa-mir-30a in NSCLC. The survival curve indicated that the low expression of hsa-mir-30a gene may shorten the survival time of patients with NSCLC. Currently, the research of hsa-mir-30a is mostly focused on bio-credit analysis, but few reports on its specific regulation mechanism. Exploring the regulatory mechanism of has-miR-30a in NSCLC would be our next research goal.

The GO term of hsa-mir-30a’s relative mRNA was mainly enriched in RNA polymerase II specificity. RNA polymerase II is related to the cell cycle. Most of the cell cycle of cancer cells is static, and a few are dynamic [26, 27]. Static cancer cells are the main obstacle to the treatment of cancer [28]. Cancer cells in a resting state will not perform transcription or other functions, resulting in RNA polymerase II to suspended [29]. The suspension of RNA polymerase II could form many DNA double-strand breaks, increase the risk of chromosome ectopic, and cause cancer [30, 31]. Zhang’s research showed that reducing RNA polymerase II in a suspended state could reduce the occurrence of tumors [32]. Therefore, we speculated that regulating the expression of hsa-mir-30a may change the abnormally quiescent cell cycle of cancer cells and inhibit the development of NSCLC. At the same time, the KEGG pathway showed that the function of hsa-mir-30a was related to cytochrome P450. Cytochrome P450 (CYP) has carcinogenic effects. Clinically, cytochrome P450 variants CYP3A and CYP2E1 could induce hepatocellular carcinoma and nasopharyngeal carcinoma, respectively [33, 34]. At the same time, P450 also participates in the metabolism and elimination of drugs, thereby reducing its pharmacological effects. The cytochrome P450 variant CYP2C8 has a unique active structure and can metabolize more than 100 drugs, mainly responsible for the metabolism of the anticancer drug Paclitaxel [35]. Therefore, by regulating the expression of cytochrome P450, the utilization rate of the metabolism of a variety of chemotherapeutic drugs can be affected. The researchers also confirmed the correlation between miR-30a-5p and tumor resistance in ovarian cancer cell lines [36]. The result proved that our prediction of the function of miR-30a-related mRNA might be correct. MiR-30a was involved in regulating the cell cycle and tumor drug resistance, and we would find new ways to treat tumors from these two aspects. Meanwhile, the problem is that we still do not know the specific therapeutic effect of hsa-mir-30a on NSCLC. We still need to verify whether extensive data analysis and prediction results are consistent with the actual situation of basic clinical experiments.

## 5 Conclusion

In this study, bioinformatics analysis was used to identify hsa-mir-30a with significant differences in expression between NSCLC samples and paracancer samples. And hsa-mir-30a may be involved in regulating the cell cycle process of NSCLC cells or drug metabolism. In the future, we would further carry out relevant basic experimental research on hsa-mir-30a, explore its regulatory mechanism, and provide new therapeutic targets for NSCLC.

## Supporting information

S1 File Source code used for bioinformatics analysis. (ZIP)
